# Endocytosis of Integrin-Binding Human Picornaviruses

**DOI:** 10.1155/2012/547530

**Published:** 2012-11-27

**Authors:** Pirjo Merilahti, Satu Koskinen, Outi Heikkilä, Eveliina Karelehto, Petri Susi

**Affiliations:** ^1^Department of Virology, University of Turku, Kiinamyllynkatu 13, 20520 Turku, Finland; ^2^Degree Program in Biotechnology and Food Technology, Turku University of Applied Sciences, Lemminkäisenkatu 30, 20520 Turku, Finland; ^3^Joint Biotechnology Laboratory, University of Turku, Tykistökatu 6a, 20520 Turku, Finland

## Abstract

Picornaviruses that infect humans form one of the largest virus groups with almost three hundred virus types. They include significant enteroviral pathogens such as rhino-, polio-, echo-, and coxsackieviruses and human parechoviruses that cause wide range of disease symptoms. Despite the economic importance of picornaviruses, there are no antivirals. More than ten cellular receptors are known to participate in picornavirus infection, but experimental evidence of their role in cellular infection has been shown for only about twenty picornavirus types. Three enterovirus types and one parechovirus have experimentally been shown to bind and use integrin receptors in cellular infection. These include coxsackievirus A9 (CV-A9), echovirus 9, and human parechovirus 1 that are among the most common and epidemic human picornaviruses and bind to **α**V-integrins via RGD motif that resides on virus capsid. In contrast, echovirus 1 (E-1) has no RGD and uses integrin **α**2**β**1 as cellular receptor. Endocytosis of CV-A9 has recently been shown to occur via a novel Arf6- and dynamin-dependent pathways, while, contrary to collagen binding, E-1 binds inactive **β**1 integrin and enters via macropinocytosis. In this paper, we review what is known about receptors and endocytosis of integrin-binding human picornaviruses.

## 1. Introduction

Picornaviruses (family *Picornaviridae*) include a diverse group of viruses, arguably best known not only as causes of devastating acute human (polio) and animal diseases (foot and mouth disease) but also by as the most common infectious disease, common cold, caused by human rhinoviruses, aseptic meningitis caused by coxsackieviruses, and more recently by severe CNS infections of newborns caused by human parechoviruses [1–4]. Clinical manifestations of picornaviruses are variable, including respiratory symptoms, gastroenteritis, rash, myocarditis, neonatal sepsis-like disease, and infections of the central nervous system such as acute flaccid paralysis, meningitis, and encephalitis [5–8]. Some virus types have also been linked to chronic diseases such as type 1 diabetes mellitus [9, 10] and wheezing illnesses that may develop into asthma [11, 12]. In spite of clinical importance of picornaviruses, there are no approved drugs against them, and the only vaccines are against three poliovirus types and hepatitis A virus [2].

Currently, there are twelve genera with 29 species in the family *Picornaviridae*. Six genera contain virus types that infect humans (*Enterovirus*, *Cardiovirus*, *Aphthovirus*, *Parechovirus*, *Hepatovirus*, and *Kobuvirus*) [13, 14]. The genus *Enterovirus *contains most of the human picornaviruses, with more than 250 recognized types, while the genus *Parechovirus *contains 16 recognized types based on VP3/VP1 sequence analysis (http://www.picornaviridae.com/). Since many of the newly discovered entero- and parechoviruses have been identified only by genetic methods [15], there is no purified virus material available for experimental studies. Thus far most picornaviral studies have been performed using a limited number of model viruses (such as poliovirus, echovirus 1, coxsackievirus B3, and human rhinovirus 2), and, hence, we know next to nothing of life cycle of other picornaviruses. Life cycle of most human viruses is dependent on efficient entry through plasma membrane into cell cytosol in endosomal vesicles, which transport the viruses to the site of replication in the cell interior. Internalization of many viruses involves classical clathrin-mediated endocytosis. However, our understanding on endocytic routes has been challenged during the past decade by introduction of novel pathways including macropinocytosis and caveolar/lipid raft-mediated endocytosis and other pathways of which we have rather limited information. During the recent years, the area of (viral) endocytosis has been the subject of extensive reviewing [16–29]. Whereas many viruses that use integrins as their cellular receptors are internalized in clathrin-coated pits and later found in endosomes [30–32], some integrin-binding picornaviruses seem to use alternative entry pathways. The aim of this paper is to give an overview of what we know about receptors and endocytosis of integrin-binding human picornaviruses. Although limited in number, they include virus types that are epidemic and pathogenic. Interestingly, these viruses share common features, which are not reflected in their receptor use and mechanism of endocytosis. Coxsackievirus A9 (CV-A9) and human parechovirus 1 possess RGD motif through which they bind *α*V-integrins. However, they seem to prefer different integrin receptors in cellular entry, and the endocytic route is also different. CV-A9 seems to endocytose via a novel Arf6- and dynamin-dependent pathway, which has not previously been described for viruses. Contrary to the expected, echovirus 1 binds inactivated integrin and is capable of alleviating the natural signaling mechanism in cell entry. Studies on virus receptors in general may give clues on viral endocytic routes and therefore enable understanding of the mechanisms of virus receptor-mediated endocytosis. This will allow the identification of the common key molecules that mediate virus life cycle and help in development of antivirals against picornaviruses.

## 2. Virus Structure, RGD Motif, and Integrin-Binding Picornaviruses

Picornaviruses are small icosahedral viruses composed of three or four capsid proteins (VP1-4 or VP0, VP1, and VP3 in the case of human parechoviruses, which lack proteolytic processing between VP4 and VP2) [33, 34]. While VP1-3 forms the icosahedral shell, VP4 protein resides in the inner surface and is probably in contact with a single species of single-stranded, plus-sense viral RNA genome, which is approximately 7000–8500 nucleotides long. RNA organization is fairly similar across the virus family [14]. A single polyprotein is produced from the RNA genome, which is proteolytically cleaved into mature structural (capsid) and nonstructural proteins. Capsid monomers assemble into pentamers, which form the complete icosahedral shell of the virus with twelve pentamer subunits. The structure of monomers and their spatial location determine the surface topology of the virus particle and are responsible for receptor binding. VP1 is the most important protein in receptor binding. Enteroviruses possess a deep depression, “canyon,” in the fivefold axis below VP1, which is involved in receptor interactions [35, 36]. The canyon has been targeted with small molecular compounds to develop antivirals, and several compounds have been shown to bind to the canyon and inhibit virus infection [37, 38]. Disappointingly, none of the compounds is in clinical use. The first structure of human parechovirus was only recently elucidated in complex with integrin receptor, and the coordinates for receptor binding were similar to enteroviruses [39]. In all, high-resolution structures of virus-receptor complexes have been determined for only a few picornaviruses, and there is a clear lack of knowledge regarding virus-receptor binding, and subsequent endocytic events.

More than ten primary cellular receptors have been implicated in picornavirus infections, and many of them belong to the immunoglobulin superfamily [2, 40, 41]. One group of such picornaviral receptors are integrins, which form a family of transmembrane glycoproteins that form noncovalent heterodimers with eighteen *α*- and eight *β*-integrin subunits that combine to form 24 *αβ* receptors [42]. Besides picornaviruses, integrins are optimal cellular receptors for adeno-, rota-, hanta-, and herpesviruses [43] because they are abundantly expressed on most cell types (see integrins at http://www.signaling-gateway.org/). In addition, integrins are connected to signalling proteins that may trigger endocytic pathways. They regulate a wide variety of cellular processes such as cell migration, growth, phagocytosis, inflammation, wound healing, and neoplasia, and they also participate in adhesive events including haemostasis and thrombosis [44]. The long extracellular domains of integrins interact with various extracellular matrix (ECM) proteins (laminin, collagen, vitronectin, and fibronectin) and other ligands including viruses, while the short cytoplasmic domains of integrins interact with the actin cytoskeleton and its components. Thus, the functional domains of integrin link the external side of the plasma membrane to the internal side of the cells to mediate internalization and endocytosis of cargo molecules [2, 45, 46]. Activation of integrin-mediated signalling is considered to be an essential mechanism for the internalization of viruses. In this process, they may mimic the natural ligands. The mechanisms regulating virus entry seem to significantly overlap with the known endocytic pathways for integrins and may, therefore, provide important insights into the mechanisms regulating integrin traffic. However, recent findings indicate that binding and internalization of some viruses such as echovirus 1 to integrin receptor differ from that of natural ligand (see below) indicating that viruses are capable of using alternate entry pathways. Integrin-binding picornaviruses also use other cell surface molecules for binding and/or entry into cells. Currently, the actual function of both integrins and these other molecules in virus infection is largely unknown, and it remains to be shown whether they are simply attachment receptors or mediators of virus internalization [2].

Out of more than one hundred enterovirus and sixteen human parechovirus types [13], there are around twenty model viruses for which there is experimental evidence for receptor binding and/or entry route [2]. Three enterovirus types (coxsackievirus A9 (CV-A9), echovirus 9 (E-9), and echovirus 1 (E-1), and a single parechovirus type (human parechovirus 1 (HPeV-1)) have been experimentally shown to bind to integrin receptors [39, 47, 48]. With the exception of E-1, they possess a specific RGD (arginine-glycine-aspartic acid) motif for integrin binding [49]. RGD-binding integrins include five *α*V integrins (*α*V*β*1, *α*V*β*3, *α*V*β*5, *α*V*β*6, and *α*V*β*8), two *β*1 integrins (*α*5*β*1 and *α*8*β*1), and *α*IIb*β*3 and share the ability to recognize cellular ligands such as fibronectin and vitronectin via RGD motif [50]. CV-A9 and HPeV-1 have been shown to bind *in vitro* to *α*V*β*3 and *α*V*β*6 [39, 47, 51], and there is experimental evidence for the binding of E-9 to integrin *α*V*β*3 [48]. In addition, there are other proteins that contribute to cellular infection of these viruses, which complicate the analysis of their role in viral endocytosis. E-1 is non-RGD enterovirus, which binds to integrin *α*2*β*1. Besides E-1, there is a single report claiming that several other non-RGD echoviruses bind to integrin *α*V*β*3 [52], but the results have not been confirmed by other groups.

## 3. *In Silico *Analysis of RGD Motif on Viral Sequences

Analysis of all picornaviral sequences in the GenBank (on 26.6.2012) with the scan for protein motif method implemented in DAMBE (http://dambe.bio.uottawa.ca/software.asp) revealed novel picornaviruses that contain RGD motif(s), and which may thus use integrin receptors in cellular entry (Table 1). RGD motifs were identified in four types of *Human enterovirus B* species and in one type of *Human enterovirus C*. There were no RGDs in the sequences of virus types in *Human enterovirus A* and *D* species. Among the eight parechovirus sequences analyzed, an RGD motif was identified in five of them (HPeV-1, 2, 4, 5, and 6). This motif was found in similar locations in the flexible C-terminus of VP1 protein in CV-A9, E-9, EV-B83, and EV-B99. Although the C-terminal 15 amino acids of VP1 containing the RGD motif could not be identified in the X-ray structure [53], the structural coordinates for integrin binding to CV-A9 and HPeV-1 suggest that this site may be functional in integrin binding, and therefore all of these viruses may bind to integrin receptors via an RGD motif [39]. Structural comparison of other putative RGD sites to the structure of human echovirus 7 (E-7; PDB id: 2x5i) with Discovery Studio software (Discovery Studio Modeling Environment, Release 3.1, San Diego: Accelrys Software Inc., 2011) suggested that they are surface-exposed. However, further experimentation is needed to elucidate their role in integrin binding. In all, there may be in total of four enteroviruses and five parechoviruses, which possess RGD motif in the C-terminus of VP1 protein and may therefore bind integrin receptor(s) (Table 1).

## 4. Mechanisms of Endocytosis of Integrin-Binding Human Picornaviruses

### 4.1. Echovirus 1

One of the most studied picornaviruses in respect to integrin receptor binding and endocytosis is echovirus 1 (E-1). While the other integrin-binding human picornaviruses bind *α*V-integrins via an RGD motif near the C-terminus of VP1, E-1 is exceptional in that it is the only known picornavirus to bind integrin *α*2*β*1 [54]. Integrin *α*2*β*1 (also known as Ia-IIa, VLA-2, ECMR-II) is a collagen receptor, which is expressed in fibroblasts, platelets, and in endothelial and epithelial cells [55]. The primary binding site of E-1 in integrin *α*2*β*1 is the inserted domain in the *α*2 subunit (*α*2I) [56–58]. The binding site of E-1 overlaps with binding site of collagen thus preventing collagen interaction with *α*2I domain when virus binds to integrin [54, 59]. E-1 binds to that domain ten times more efficiently than collagen [54]. In a cryo-EM analysis, the virus particle appeared to be decorated with 60 copies of the integrin *α*2I domain suggesting that each VP1-3 protomer can bind one *α*2I domain. The *α*2I domain interacts with the VP2 of one protomer and the VP3 of a neighbouring protomer in the E-1 capsid [54]. Binding of E-1 to integrin *α*2*β*1 does not induce uncoating but instead may lead to the stabilization of capsid suggesting that viral RNA is released during endocytosis and not on plasma membrane [37, 46]. Unlike the cellular ligand, collagen, which binds active, extended form of the receptor with subsequent elicitation of integrin-mediated signalling cascades and endocytosis, E-1 was recently shown to prefer bent form of *α*2*β*1 in binding [60, 61]. Virus binding does not lead to activation of the mechanism of cellular endocytosis but instead the virus clusters integrins, which seems to be essential for rapid internalization of the virus [60, 61]. Thus, E-1 is targeted to a novel, *β*1-mediated endocytic pathway, but the mechanism remains unclear.

 The initial observations of cellular entry of E-1 suggested that the virus uses caveolar/raft-dependent entry pathway. This was based on the virus accumulation in caveolin-1-positive endosomes in SAOS cells overexpressing integrin *α*2*β*1 [45, 46]. However, at the same time and using another cell model, CV-1, the same authors demonstrated that majority of E-1 do not colocalize with caveolin-1 on the plasma membrane [54]. This observation was based on parallel comparisons to SV40, which is known to use caveolar route at least in some cell lines [47]. However, it is now evident that while some E-1 particles accumulate in caveolin-1-positive vesicles, the internalization mechanism of E-1 has features of macropinocytosis since many signalling molecules that are involved in E-1 infection are macropinocytosis-linked [16, 62]. These molecules include PKC*α*, PLC, PI3 K, Rac1, Pak1, and CtBP1/BARS [60, 61]. Caveolar pathway was originally characterized by structural proteins of caveosomes, caveolin-1, and caveolin-2 and lacks typical markers of clathrin-mediated endocytosis such as epsin family proteins and clathrin. Dynamin has been shown to be indispensable in caveolar pathway [23, 63]. However, it has been suggested that caveolar, lipid raft-dependent, and caveolin-1-independent endocytic pathways have the same underlying mechanisms [64]. In addition, the view on caveosomes has recently been revised in that they correspond to modified late endosomes or endolysosomes, and it has been suggested that the term caveosome is no longer to be used [65].

More recent studies indicate that in the early stage of endocytosis, E-1 is found in tubulovesicular structures, which contain fluid-phase markers but do not contain caveolin-1, GPI-anchored proteins, or flotillin. This suggests that E-1 entry pathway originates from lipid rafts and is not linked to caveolar or flotillin pathways. Studies on *α*2-positive structures indicated that *α*2/E-1 is internalized in pH-neutral vesicular bodies, which lack the features of clathrin-mediated endocytosis. Yet, E-1 was also detected in caveolin-1-positive structures after 15 minutes postinfection [66–68] and dominant-negative caveolin-3 has been shown to block E-1 infection [52]. These data suggest that caveolar pathway is somehow linked to E-1 endocytosis. The novel structures that contain E-1 and internalized integrins are called *α*2 integrin containing multivesicular bodies (*α*2-MVBs) [60, 61, 68, 69]. The *α*2-MVBs are degradative structures distinct from lysosomes, autophagosomes, and proteasomes [69, 70]. The nature of *α*2-MVBs involved in E-1 entry was confirmed by using immunofluorescence and siRNAs against components of endosomal sorting complex of transport proteins (ESCRTs), which are localized in early endosomes and function in MVB formation [55]. Their role in E-1 endocytosis is still unclear but they may prevent the contact between E-1 and lysosomal hydrolases, which would be detrimental for successful E-1 infection [69, 70]. The recent finding that ESCRT complex recruits caveolin-1 into maturing intralumenal vesicles may explain why E-1 and caveolin-1 are found in similar structures early in infection [46]. After internalization, clustered integrins are not recycled back to plasma membrane in strict contrast to slow recycling of unclustered *α*2 integrins [70]. This may indicate that E-1 has means to reroute endocytic vesicles, which contain *α*2 integrins. Internalization of *β*1 integrin has been shown to be regulated by Rab5/Rab21 and microtubules [43] as well as active Arf-6 [71]. However, the role of Arf-6 in viral endocytosis ([72]; see below) was only recently demonstrated, and such studies have not been performed with E-1.

### 4.2. Echovirus 9 and Coxsackievirus A9

Echovirus 9 (E-9) and coxsackievirus A9 (CV-A9) are structurally very similar viruses. They possess a receptor-binding motif, RGD, at the C-terminus of the VP1 protein through which they interact with cell surface integrin(s). They are epidemic viruses and among the most common etiological agents of aseptic meningitis [73–76]. Two known strains of E-9, Barty and Hill, differ in that Barty strain possesses RGD motif while Hill does not. The lack of RGD severely affects the infectivity of the Hill strain [48], and it has been speculated whether Hill is a true strain or instead, a recombinant form between Barty strain and E-18 [77]. RGDless E-9 viruses have not been detected in epidemiological samples, and, therefore, it is likely that RGD motif for integrin binding is imperative for successful E-9 infection [48]. Since there are no imaging studies on endocytosis of E-9, it remains to be determined whether E-9 follows the same route as CV-A9 since the viruses are very similar in sequence and pathogenicity [53]. With this in mind, the remaining part of this chapter will focus on receptors and endocytosis of CV-A9.

Although a number of cell surface molecules have been proposed to contribute to CV-A9 infection, the internalization mechanism that follows after the virus particle has bound to cell surface is still poorly characterized. CV-A9 has been shown to interact *in vitro* with integrin *α*V*β*3 and *α*V*β*6 via RGD motif [51, 78, 79]. RGD motif seems to be essential in clinical CV-A9 infections since it is conserved in CV-A9 isolates [80]. However, Hughes et al. [81] and Roivainen et al. [82] showed that artificial CV-A9 mutants lacking the RGD motif efficiently infected human rhabdomyosarcoma (RD) cells, indicating that there are also an RGD-independent attachment and an internalization mechanism. In addition, there are cell lines that do not express *α*V-integrins but which are highly susceptible to infection with CV-A9 [83]. Triantafilou et al. [84] showed that CV-A9 binds to Chinese hamster ovary (CHO) cells that overexpress human integrin *α*V*β*3, although this interaction does not lead to productive cell infection. More recently, it has been shown that integrin *α*V*β*6 binds with higher affinity to CV-A9 than integrin *α*V*β*3 suggesting that it is the primary cellular receptor for CV-A9 [47]. The binding to *α*V*β*6 was recently shown to occur at nanomolar affinity but there were no indications of structural changes that would lead to RNA release [85]. Besides integrin(s), there are other cellular molecules that participate in the entry stage of CV-A9 infection. Triantafilou et al. [84] were the first to demonstrate that antibody against *β*2-microglobulin (a subunit of major histocompatibility complex class I (MHC-I) complex *β*2M) inhibited CV-A9 infection completely in susceptible cell lines. In addition, heat shock 70-kDa protein 5 (HSPA5; also known as glucose-regulated protein 78-kDa, or GRP78) has been shown to mediate CV-A9 infection possibly via its interaction with *β*2M [86]. Recent report suggests that some CV-A9 isolates bind heparan sulphate [87].

Endocytosis of CV-A9 has mainly been followed in green monkey kidney (GMK) and human lung carcinoma (A549) cell lines [72, 88, 89]. Hecker et al. [88] demonstrated in their early work by electron microscopy that CV-A9 particles enter GMK cells in vesicles, which then occasionally fuse and form larger structures. It was also found that most internalized virus particles became trapped in large vacuoles (presumably lysosomes) where they were confined without proceeding to capsid uncoating and RNA release. In GMK cells, the entry of CV-A9 was proposed to occur through lipid microdomains where a number of signalling events takes place [89]. Other enteroviruses, for example E-1 [66, 67] and E-11 [90], have also been shown to utilize lipid microdomains for cellular entry. However, lipid rafts are involved in several entry pathways and, therefore, are not very indicative of cell entry route [20]. A more detailed study on CV-A9 endocytosis was conducted in A549 cells using siRNAs and confocal imaging [72]. Contrary to expected, silencing of Src, Fyn, RhoA, PI(3)K, and Akt1 that have been linked to integrin signalling had no effect on CV-A9 infection. In addition, CV-A9 did not cause integrin receptor clustering as in the case of E-1 and *α*2*β*1. We recently showed [72] that CV-A9 internalization is dependent on *β*2-microglobulin [75]. CV-A9 was internalized but retained at the cell periphery following the silencing of *β*2M. This was the first visual demonstration that *β*2M functions at postinternalization stage. These data suggest that integrin *α*V*β*6 acts merely as binding receptor while the internalization is mediated by *β*2M.

The current view on virus endocytosis is based on specific cellular markers that are used to distinguish between the endocytic routes. However, many central markers have been found to overlap between these routes. For example, dynamin was originally noted for its role in severing clathrin-coated vesicles from plasma membrane, but was then found to be indispensable for example in caveolar and some other clathrin-independent pathways [24, 71, 91]. The role of Arf6 in virus endocytosis is also becoming increasingly important. Arf6 (ADP-ribosylation factor 6) is a small GTPase, which has multiple roles in the regulation of membrane traffic and other cellular functions, but it was only recently when it was linked to virus endocytosis [75]. Although it has been suggested in the current classification schemes that Arf6-dependent entry pathway is dynamin-independent [20], it has been shown that structural protein VP22 of Herpes simplex virus uses both Arf6 and dynamin in the internalization process in HeLa cells. The entry process of HSV-VP22 appears to be similar to that of CV-A9 since internalization of HSV-VP22 is independent of clathrin, caveolin, and the Rho family GTPases RhoA, Rac1, and Cdc42 [92]. Our recent data indicate that CV-A9 entry, irrespective of RGD, is dependent on *β*2M, HSPA5, dynamin, and Arf6, and that CV-A9 and Arf6 are coendocytosed, that is, they are localized in the same vesicles when being transported from cell surface to the interior [72, 83, 84, 86]. Interestingly, Arf6 also regulates the endocytosis of MHC-I proteins [93] including *β*2M, which suggests a possible mechanistic link between these molecules and therefore internalization of CV-A9.

One of the many unanswered questions regarding CV-A9 infection is the role of integrin and other putative receptors play in cellular entry. Conceptually, there are attachment receptors that bind viruses to concentrate them on the cell surface, and actual virus receptors, which in addition to their role as binding receptors trigger changes in the virus structure to potentiate RNA release and induction of cellular signalling that mediates virus internalization. It is evident that integrin *α*V*β*6 serves as high-affinity binding receptor for CV-A9, while at least two other molecules mediate the internalization and possibly cellular endocytosis of the virus in cell-specific manner. In a simplified model, CV-A9 particle binds first to the cell surface integrin *α*V*β*6 (in RGD-dependent endocytosis) and then rapidly forms association with *β*2M (and HSPA5). Following this, the virus is endocytosed via an Arf6-mediated route, perhaps still in association with *β*2M. However, the conserved nature of RGD motif in clinical CV-A9 isolates indicates that integrin binding must have importance in multicellular infection. It is possible that CV-A9 is capable of altering receptor use at organism level while maintaining its infectivity by existing in quasispecies form, and this may explain why it remains highly pathogenic [63]. There are also reports claiming that *β*2M participates in entry stage of several echoviruses including E-1 [56, 94] and possibly human parechovirus 1 (see below). The MHC I/*β*2M is linked to integrin-mediated cellular endocytosis and may serve as cue to reroute viruses into novel locations within endosomal network. Endocytosis of MHC I has been studied in detail in HeLa cells, and it is visible in endosomes that are distinct from those containing cargo, such as transferrin receptor that enters by clathrin-mediated endocytosis. At later time points (10–15 min), MHC I is observed in classical early endosomes containing the transferrin receptor, Rab5, and the early endosomal antigen 1 (EEA1). EEA1 is a protein that is recruited to membranes by phosphatidylinositol 3-phosphate (PI3P) and Rab5-GTP, and together these molecules facilitate early endosome fusion. From there MHC I can be either routed to late endosomes for degradation [95] or recycled back to plasma membrane [93, 95, 96]. Recycling of MHC I back to the plasma membrane requires the activity of Arf6. Thus, the possible involvement of *β*2M in infectious entry of many picornaviruses suggests that such viruses may also use Arf6-mediated entry pathway, and therefore Arf6 may be a common entry pathway for many picornaviruses.

### 4.3. Human Parechovirus 1

Currently, there are 16 human parechovirus types in the genus *Parechovirus* [3]. Parechovirus infections are common during the first years of life and often asymptomatic or related to mild gastroenteritis and respiratory infections [6, 97–100]. However, HPeV-1 and HPeV-3 may cause severe illnesses such as infections of the central nervous system, generalized infections of neonates and myocarditis [3, 97, 101]. HPeV-1, -2, -4, -5 and -6 possess an RGD motif near the C-terminus of VP1 that is known to facilitate binding of cellular ligands to integrins (Table 1) [33, 102]. HPeV-1 has been shown to interact with integrin *α*V*β*1, *α*V*β*3, and *α*V*β*6 [39, 103–105]. In contrast to CV-A9, RGD motif is essential for viability of HPeV-1; mutation in the RGD motif resulted in noninfectious phenotype, and only viruses in which the RGD sequence had been restored by reversion recovered after cell passage [106].

Although there are similarities in receptor use *in vitro* between CV-A9 and HPeV-1, the viruses seem to use different endocytic pathways. Both viruses bind to integrins *α*V*β*3 and *α*V*β*6 *in vitro*, but possess higher motif-to-motif affinity to integrin *α*V*β*6 than to *α*V*β*3. Cryo-EM studies have shown that *α*V*β*6 binds with high affinity to surface of HPeV-1 via RGD loop located in the C terminus of VP1 [39]. In contrast, *β*2M, which has been suggested to play role in E-1 and CV-A9 infections [72, 84, 94], may not be significant in HPeV-1 infection [105]. Despite the receptor use CV-A9 and HPeV-1 seem to be endocytosed in different manner. There is a single publication on HPeV-1 endocytosis (or endocytosis of any parechovirus), and this paper suggests that HPeV-1 uses clathrin-mediated endocytic pathway [105]. Following internalization, HPeV-1 enters early endosomes after which the virus is found in late endosomes [105]. The recent findings from our laboratory indicate that HPeV-1 binds preferably integrin *β*1 and that *β*2M participates in the entry process (P. Merilahti and P. Susi, manuscript in preparation). It has been suggested that the clustering and activation of *β*1-integrins trigger caveolar endocytosis with the subsequent removal of *β*1-integrins from the plasma membrane [43, 107], which is evidently in contradiction with the suggestion that HPeV-1 is endocytosed via clathrin-mediated pathway [88]. On the other hand, MHC I (with *β*2M) has been linked to internalization of *β*1-integrins, but previously not shown to be involved in HPeV-1 infection [88]. It has also been demonstrated that Arf6 regulates endocytosis of *β*1 [108], but this has not been demonstrated for parechoviruses. Thus, further studies are needed to elucidate the role of integrin *β*1, involvement of *β*2M, and Arf6-pathway in parechovirus infection.

## 5. Conclusions

Although there has been means to cultivate picornaviruses since the discovery of cell culture methods in the 1950s and despite the economic importance of picornaviruses, there are still no drugs in clinical use against any picornavirus type. This is partially because of the large number of picornavirus types that infect humans, the complex nature of the infection process, and the lack of knowledge of receptor tropism and mechanisms of endocytosis. Experiments have been conducted with three pathogenic picornavirus types (CV-A9, E-9, and HPeV-1) that possess RGD motif for integrin binding. In addition, there are other human picornaviruses that possess RGD motif and may therefore use integrin(s) and cellular receptor(s) (Table 1). Yet, another integrin-binding human picornavirus, echovirus 1 (E-1), is exceptional in binding the RGD-independent integrin *α*2*β*1. Recent studies on these viruses have shown surprisingly that whereas CV-A9 uses novel HSPA5 receptor and Arf6- and dynamin-dependent entry route in infection, cellular entry of E-1 alleviates the route of the natural ligand in integrin *β*1; E-1 binds inactive receptor form, and internalization is dependent on clustering and not on integrin-mediated signals. These are examples of assignment of picornaviruses to novel entry mechanisms that have not previously been described for viruses, and which differ from cellular endocytosis. Since most of the cellular studies on receptor binding and endocytosis have been conducted in cancer cell models, it remains to be determined what the relevance of detected receptor interactions and mechanisms of endocytosis in native human cells and tissues, and in model organisms, is. The future issues in such studies include the mechanism of endocytosis between viruses and natural ligands that bind the same receptor, the possible central role of *β*2M, the possible involvement of Arf6 pathway, and the role of novel receptors and mediators such as HSPA5 in picornavirus infection. Studies on mechanisms and mediators of endocytosis and comparisons to other picornaviruses are likely to be useful in designing receptor-targeted or intracellular drugs with broad specificities. Large-scale genomic screens and clustering analysis may also reveal common features and nominators that may be useful in drug discovery against picornaviruses in general [109]. Existing therapeutic agents against integrins may also prove to be useful as antivirals against integrin-binding human picornaviruses [110].

## Figures and Tables

**Table 1:** RGD motif(s) identified in picornaviral capsid proteins.

Type	Acc no.	Location of RGD motif in viral polyprotein	
		VP3	VP1
CV-A9	D00627		858–860
E-5	AF083069	410–412	
E-9	X84981		651–653/860–862
EV-B83	AY843301		832–834
EV-B99	JF260926		860–862
HPeV-1	L02971		764–766
HPeV-2	AJ005695		763–765
HPeV-4	DQ315670		767–769
HPeV-5	AF055846		772–774
HPeV-6	AB252582		766–768
