# Erratum to “Endocytosis of Integrin-Binding Human Picornaviruses”

**DOI:** 10.1155/2013/412909

**Published:** 2013-04-03

**Authors:** Pirjo Merilahti, Satu Koskinen, Outi Heikkilä, Eveliina Karelehto, Petri Susi

**Affiliations:** ^1^Department of Virology, University of Turku, Kiinamyllynkatu 13, 20520 Turku, Finland; ^2^Degree Program in Biotechnology and Food Technology, Turku University of Applied Sciences, Lemminkäisenkatu 30, 20520 Turku, Finland; ^3^Joint Biotechnology Laboratory, University of Turku, Tykistökatu 6a, 20520 Turku, Finland

Due to unfortunate errors at the proof-reading stage, there are several misplaced references. A list of correct references in specified sentences is provided here as follows.

Page 3: binding of E-1 to integrin **α**2**β**1 does not induce uncoating but instead may lead to the stabilization of capsid suggesting that viral RNA is released during endocytosis and not on plasma membrane [54, 60].

Page 3: this was based on the virus accumulation in caveolin-1-positive endosomes in SAOS cells overexpressing integrin *α*2*β*1 [60, 66]. However, at the same time and using another cell model, CV-1, the same authors demonstrated that majority of E-1 do not colocalize with caveolin-1 on the plasma membrane [67]. This observation was based on parallel comparisons to SV40, which is known to use caveolar route at least in some cell lines [62].

Page 4: dominant-negative caveolin-3 has been shown to block E-1 infection [68].

Page 4: which are localized in early endosomes and function in MVB formation [69].

Page 4: the recent finding that ESCRT complex recruits caveolin-1 into maturing intralumenal vesicles may explain why E-1 and caveolin-1 are found in similar structures early in infection [66, 69].

Page 5: we recently showed that CV-A9 internalization is dependent on *β*2-microglobulin [72].

Page 5: Arf6 (ADP-ribosylation factor 6) is a small GTPase, which has multiple roles in the regulation of membrane traffic and other cellular functions, but it was only recently when it was linked to virus endocytosis [72].

Page 5: and this may explain why it remains highly pathogenic [75, 76].

Page 5: which is evidently in contradiction with the suggestion that HPeV-1 is endocytosed via clathrin-mediated pathway [105]. On the other hand, MHC I (with *β*2M) has been linked to internalization of **β**1-integrins, but previously not shown to be involved in HPeV-1 infection [105].

Page 9: the data in reference [83] should be as follows: O. Heikkilä, E. Karelehto, P. Merilahti et al., “HSPA5 protein (GRP78) and b2-microglobulin mediate internalization and entry of coxsackievirus A9 via a novel Arf6-dependent entry pathway in human epithelial colon adenocarcinoma cells,” Submitted.

